# National Analysis of Adverse Events Involving Distal Radius Plate Systems Reported to the United States Food and Drug Administration

**DOI:** 10.7759/cureus.99721

**Published:** 2025-12-20

**Authors:** Muzamil Ahmad, Maya Nassif, Annabella Jensen, Isabella Strickler, Andres S Espinoza, Leonardo M Cavinatto

**Affiliations:** 1 Department of Foundational Medical Studies, Oakland University William Beaumont School of Medicine, Rochester, USA; 2 Department of Orthopedic Surgery, Corewell Health William Beaumont University Hospital, Royal Oak, USA

**Keywords:** adverse event, device failure, maude database, prosthesis and implants, wrist fractures

## Abstract

Purpose: Distal radius plate (DRP) systems are the standard approach for unstable distal radius fractures, yet implant complications persist. We characterized the distribution of nationally reported device and patient adverse events by manufacturer, locking mechanism, and plate subtype using the United States Food and Drug Administration’s (FDA) Manufacturer and User Facility Device Experience (MAUDE) database.

Methods: We retrospectively queried the MAUDE database for DRP-related reports using product code “HRS” (Plate, Fixation, Bone) and the term “distal radius” between January 2018 and December 2024. Follow-ups and duplicates were collapsed or removed. Device and patient problem codes were mapped to categories defined a priori. Primary outcomes included overall adverse event distribution. Secondary outcomes were stratified by manufacturer, locking mechanism (fixed vs. variable-angle), and plate subtype. Categorical comparisons were made using chi-square tests with significance defined as p<0.05.

Results: We analyzed 582 unique reports containing 414 device-related and 520 patient-related codes. Leading device events were hardware breakage (32.6%), incompatibility/mismatch (17.9%), stability problems (13.3%), and installation/positioning failures (11.1%). Leading patient events were pain/discomfort (18.8%), peri-implant fracture (14.4%), loss of range of motion (ROM)/implant failure (12.3%), and soft-tissue injury (9.0%). By manufacturer (share of MAUDE reports in this sample: DePuy Synthes: 67.2%, Arthrex: 10.0%, Stryker: 9.8%, and Smith+Nephew: 5.7%), significant differences were observed for hardware breakage (p=0.004), incompatibility (p<0.001), installation/positioning (p<0.001), stability (p<0.001), device embedment/calcification (p<0.001), loss of ROM/implant failure (p=0.027), nerve/neurologic complications (p=0.010), pain/discomfort (p=0.001), patient reaction (p=0.002), and peri-implant fracture (p=0.015). Locking mechanism stratification identified a difference in the distribution of nerve/neurologic complication codes, with a higher proportion among reports classified as fixed-angle (p<0.001). Significant differences were observed across plate subtypes for hardware breakage (p=0.006), incompatibility (p=0.009), installation/positioning (p<0.001), and infection (p<0.001).

Conclusion: Hardware breakage and pain were the most frequently reported device and patient events, respectively. The distribution of reported problem-code categories varied across manufacturers, locking mechanisms, and plate subtypes. However, the observed findings are descriptive and hypothesis-generating, as MAUDE lacks exposure denominators and key clinical context, and therefore should not be interpreted as evidence of causal, comparative safety, or performance, or as recommendations for or against any specific manufacturer. Future studies with exposure data are warranted to validate these signals and evaluate comparative risks. Despite the inherent limitations of the MAUDE database, these findings present invaluable information on the reported adverse events of DRP systems and may inform proactive postoperative surveillance.

## Introduction

Distal radius fractures remain one of the most common fractures of the upper extremity [1]. While nonsurgical interventions may be pursued in select cases, open reduction and internal fixation (ORIF) using distal radius plate (DRP) systems is the standard of care for unstable distal radius fractures [2]. ORIF surgical interventions have demonstrated improved functional outcomes and reduced long-term morbidity and impairment [3,4].

Implant technological advancements such as volar and dorsal plates, fixed-angle and variable-angle locking mechanisms, and fragment-specific devices have enhanced the adaptability of DRPs, allowing them to better accommodate varying fracture patterns, patient anatomy, and clinical demands [5-8].

Despite the versatility of DRP systems, complications persist. Implant complications such as hardware breakage and fracture, stability issues, and material problems, as well as patient complications such as pain and discomfort, peri-implant fractures, infection, and soft tissue injury have all been reported [9-11]. While existing studies have assessed complications associated with DRP systems, they often use institution-specific data or report individual cases, leaving a substantial gap in our understanding of national trends by manufacturer, locking mechanism, and device subtype.

Therefore, we queried the United States Food and Drug Administration’s (FDA) Manufacturer and User Facility Device Experience (MAUDE) database, a national repository of reported medical device adverse events. The MAUDE database has previously been used to evaluate device and patient-related complications of medical devices across multiple medical specialties, expanding our current understanding of medical device complications [12-14]. This paper aims to use the MAUDE database to evaluate the reported complications of DRP systems while stratifying these reports by manufacturer, locking mechanism, and device subtype. Because MAUDE is a passive surveillance system without device-use denominators and with limited clinical detail (such as fracture pattern, surgeon technique, or surgeon experience), manufacturer and design-stratified findings in this study aim to describe reporting patterns to inform proactive surveillance, rather than assign comparative risk or causality.

## Materials and methods

Ethics statement

Due to the public, de-identified data used, this study did not constitute human subjects research and was exempt from institutional review board approval.

Study design

We performed a retrospective analysis of adverse events involving DRP systems reported to the FDA’s MAUDE database between January 1, 2018 and December 31, 2024. Reports were extracted using FDA product code “HRS” (Plate, Fixation, Bone) and query term “distal radius.”

Each report contained the following data points: medical device report (MDR) key, report number, date received, event date, event type, device problem codes, patient problem codes, device name, manufacturer family, and narrative text. To avoid repeat counting, manufacturer follow-ups were collapsed into their respective originating MDR when two or more reports shared the same MDR report number or key. Duplicate reports were removed by screening for matching MDR report numbers and keys. Reports with device and/or patient codes that contained missing information were excluded from analysis. A single report may include more than one device and/or patient code. Manufacturer identifiers were extracted from MAUDE report fields as listed in the public database and may encompass multiple product lines or plate designs within the respective parent company. All data extracted from the MAUDE database are publicly accessible and publicly reported. Manufacturers are presented solely to describe the publicly reported MAUDE entries, not to attribute fault, infer causality, or imply brand superiority/inferiority.

The primary outcome was the distribution of device-related and patient-related adverse events. Secondary outcomes stratified adverse events by manufacturer, locking mechanism, and plate subtype. As MAUDE terminology is highly variable and includes terms that partially overlap (i.e., “fracture,” “crack,” and “break”), semantically similar codes were collapsed into broader pre-defined categories for improved interpretability (Tables 1, 2). When a code was not clearly alignable with a prespecified category, it was assigned to other/unspecified.

**Table 1:** Categorized device complications and counts MAUDE, Manufacturer and User Facility Device Experience.

Device category	MAUDE term	N
Hardware breakage	Break	117
	Fracture	14
	Crack	4
Incompatibility mismatch	Device-device incompatibility	67
	Patient-device incompatibility	4
	Inadequacy of device shape and/or size	2
	Patient-device interaction problem	1
Stability issue	Unintended movement	14
	Migration	14
	Device dislodged or dislocated	6
	Device slipped	5
	Detachment of device or device component	4
	Entrapment of device	3
	Loosening of implant not related to bone ingrowth	3
	Separation problem	2
	Loose or intermittent connection	2
	Migration or expulsion of device	2
Installation/positioning	Positioning failure	16
	Fitting problem	6
	Failure to align	6
	Incomplete or inadequate connection	5
	Positioning problem	5
	Connection problem	5
	Failure to adhere or bond	2
	Malposition of device	1
Material problem	Material deformation	13
	Material twisted/bent	13
	Material fragmentation	5
	Bent	3
	Material protrusion/extrusion	1
	Deformation due to compressive stress	1
	Material puncture/hole	1
Use of device issue	Use of device problem	15
	Device operates differently than expected	8
	Off-label use	5
	Improper or incorrect procedure or method	3
	Device difficult to maintain	2
	Misassembled during installation	1
	Difficult to insert	1
	Difficult to position	1
Defective device	Defective device	11
	Mechanical problem	5
Difficulty advancing	Difficult to advance	3
	Physical resistance/sticking	2
	Failure to advance	2
Miscellaneous	Therapeutic or diagnostic output failure	2
	Contamination/decontamination problem	1
	Device damaged by another device	1
	Manufacturing, packaging, or shipping problem	1
	Flaked	1
	Mechanics altered	1
	Damaged thread	1

**Table 2:** Categorized patient complications and counts MAUDE, Manufacturer and User Facility Device Experience.

Patient category	MAUDE term	N
Pain or discomfort	Pain	76
	Discomfort	15
	Implant pain	7
Peri-implant fracture	Nonunion/delayed-union bone fracture	42
	Bone fracture(s)	32
	Limb fracture	1
Loss of range of motion or implant failure	Failure of the implant	47
	Loss of range of motion	17
Soft-tissue injury	Muscle/tendon damage	19
	Injury	14
	Unspecified tissue injury	8
	Rupture	5
	Laceration(s)	1
Healing/recovery issue	Impaired healing	14
	Swelling	3
	Malunion of bone	3
	Wound dehiscence	3
	Scar tissue	3
	Perforation	3
	Inflammation	2
	Unspecified musculoskeletal problem	2
	Ambulation or postural difficulties	1
	Fatigue	1
	Seroma	1
	Synovitis	1
	Weakness	1
	Cyst(s)	1
	Adhesion(s)	1
Patient reaction	Foreign body in patient	7
	Hypersensitivity/allergic reaction	6
	Rash	5
	Reaction	5
	Skin inflammation/irritation	4
	Skin irritation	2
	Irritation	1
	Itching sensation	1
	Erythema	1
Nerve/neurologic complication	Nerve damage	19
	Burning sensation	4
	Neuropathy	4
	Hypoesthesia	2
	Numbness	2
Infection	Unspecified infection	23
	Postoperative wound infection	4
	Abscess	1
Device embedment/calcification	Device embedded in tissue or plaque	12
	Calcium deposits/calcification	2
Vascular complication	Necrosis	2
	Blood loss	1
	Swelling/edema	1
Device issue with no consequence to patient	No consequences or impact on patient	32
	No known impact or consequence to patient	31
Miscellaneous	Physical asymmetry	7
	Fall	5
	Arthritis	4
	Joint dislocation	3
	Joint disorder	1
	Osteopenia/osteoporosis	1
	Electrolyte imbalance	1
	Autoimmune disorder	1
	Connective tissue disease	1

Device categories included hardware breakage, incompatibility mismatch, stability issue, installation/positioning, material problem, use of device issue, defective device, difficulty advancing, and miscellaneous.

Patient categories included pain or discomfort, peri-implant fracture, loss of range of motion (ROM) or implant failure, soft-tissue injury, healing/recovery issue, patient reaction, nerve/neurologic complication, infection, device embedment/calcification, vascular complication, and miscellaneous.

Statistical analysis

Microsoft Excel (Microsoft Corporation, Redmond, WA, USA) was used for descriptive statistical analyses, while Python version 3.13.1 (Python Software Foundation, Wilmington, DE, USA) was used to perform quantitative analyses. For categorical variables, Pearson chi-square (χ²) tests were used to evaluate heterogeneity in the distribution of reported problem-code categories across strata and did not estimate complication rates or comparative device risk due to the lack of exposure denominators and key confounders. A p-value of less than 0.05 was considered statistically significant.

## Results

After excluding duplicates and follow-ups, 582 unique reports were analyzed, including 414 device-related codes and 520 patient-related codes. Annual report counts from 2018 to 2024 were 105, 79, 98, 81, 74, 65, and 80, respectively.

Fifteen manufacturers were identified, with four being unspecified. DePuy Synthes accounted for 67.2% of reports in our sample (n=391), followed by Arthrex 10.0% (n=58), Stryker 9.8% (n=57), Smith+Nephew 5.7% (n=33), Acumed 2.9% (n=17), and Zimmer Biomet 1.4% (n=8). Regarding device characteristics, 70 reports involved fixed-angle systems and 285 involved variable-angle systems (the remainder being unspecified). Plate orientation was volar in 233 and dorsal in 15 (the remainder being unspecified). By device subtype, 236 reports had identifiable descriptors, with the remainder being unspecified. Column-based devices comprised 47.9% of reports (n=113), narrow 24.6% (n=58), extended/long 6.8% (n=16), and short 6.8% (n=16).

Device adverse events

Among the total reported device-related complications (n=414), the most frequent was hardware breakage (135; 32.6%), followed by incompatibility mismatches (74; 17.9%), stability problems (55; 13.3%), installation/positioning failures (46; 11.1%), material problems (37; 8.9%), use of device problems (36; 8.7%), defects/mechanical issues (16; 3.9%), and difficulty advancing the device (7; 1.7%). Less common device issues are listed in Table 1.

Device events by manufacturer

DePuy Synthes (n=253) reports were led by hardware breakage (96; 38.0%), incompatibility (66; 26.1%), stability issues (29; 11.4%), material problems (22; 8.8%), and use of device problems (22; 8.8%). Arthrex (n=58) devices most frequently reported hardware breakage (13; 22.4%), followed by installation/positioning problems (12; 20.7%) and use of device problems (8; 13.7%). Stryker (n=42) most often reported hardware breakage (15; 35.7%), followed by stability issues (8; 19.0%) and material problems (8; 19.0%). Smith+Nephew (n=34) reports were led by installation/positioning issues (21; 61.7%), hardware breakage (4; 11.8%), and defective devices (3; 8.8%). Additional categories appear in Table 3.

**Table 3:** Manufacturer distribution of device and patient problem categories Values are presented as N (%). For device category rows, percentages are calculated out of all device-related problems for each respective manufacturer (Acumed n=7; Arthrex n=58; DePuy Synthes n=253; Smith+Nephew n=34; Stryker n=42; Zimmer Biomet n=2; and other n=18). For patient category rows, percentages are calculated out of all patient-related problems for each respective manufacturer (Acumed n=13; Arthrex n=41; DePuy Synthes n=353; Smith+Nephew n=24; Stryker n=63; Zimmer Biomet n=11; other n=15). χ² (df=6) and p-values are derived via Pearson chi-square tests, representing the distributions and heterogeneity in reported problem-code categories within MAUDE. They should not be interpreted as comparative complication rates, manufacturer fault, or device superiority/inferiority.

	Acumed	Arthrex	DePuy Synthes	Smith+Nephew	Stryker	Zimmer Biomet	Other	χ²	p
Device category, N (%)									
Defective device	0 (0.0%)	4 (6.9%)	8 (3.2%)	3 (8.8%)	1 (2.4%)	0 (0.0%)	0 (0.0%)	5.36	0.499
Difficulty advancing	0 (0.0%)	2 (3.4%)	3 (1.2%)	0 (0.0%)	0 (0.0%)	0 (0.0%)	0 (0.0%)	3.70	0.717
Hardware breakage	4 (57.1%)	13 (22.4%)	96 (37.9%)	4 (11.8%)	15 (35.7%)	1 (50.0%)	2 (11.1%)	18.9	0.004
Incompatibility mismatch	1 (14.3%)	5 (8.6%)	66 (26.1%)	0 (0.0%)	0 (0.0%)	0 (0.0%)	2 (11.1%)	32.6	<0.001
Installation/positioning	0 (0.0%)	12 (20.7%)	7 (2.8%)	21 (61.8%)	6 (14.3%)	1 (50.0%)	0 (0.0%)	115.9	<0.001
Material problem	0 (0.0%)	4 (6.9%)	22 (8.7%)	2 (5.9%)	8 (19.0%)	0 (0.0%)	1 (5.6%)	7.12	0.31
Miscellaneous	0 (0.0%)	3 (5.2%)	0 (0.0%)	3 (8.8%)	1 (2.4%)	0 (0.0%)	1 (5.6%)	18.2	0.005
Stability issue	0 (0.0%)	7 (12.1%)	29 (11.5%)	1 (2.9%)	8 (19.0%)	0 (0.0%)	10 (55.6%)	34.5	<0.001
Use of device problem	2 (28.6%)	8 (13.8%)	22 (8.7%)	0 (0.0%)	3 (7.1%)	0 (0.0%)	2 (11.1%)	8.82	0.184
Total	7 (100.0%)	58 (100.0%)	253 (100.0%)	34 (100.0%)	42 (100.0%)	2 (100.0%)	18 (100.0%)	-	-
Patient category, N (%)									
Device embedment/calcification	0 (0.0%)	9 (22.0%)	2 (0.6%)	2 (8.3%)	1 (1.6%)	0 (0.0%)	0 (0.0%)	68.4	<0.001
Device issue with no consequence to patient	2 (15.4%)	0 (0.0%)	42 (11.9%)	6 (25.0%)	13 (20.6%)	0 (0.0%)	0 (0.0%)	17.4	0.008
Healing/recovery issue	0 (0.0%)	5 (12.2%)	22 (6.2%)	3 (12.5%)	7 (11.1%)	0 (0.0%)	3 (20.0%)	9.25	0.16
Infection	0 (0.0%)	0 (0.0%)	27 (7.6%)	0 (0.0%)	0 (0.0%)	0 (0.0%)	1 (6.7%)	12.3	0.057
Loss of range of motion or implant failure	1 (7.7%)	8 (19.5%)	34 (9.6%)	4 (16.7%)	15 (23.8%)	0 (0.0%)	2 (13.3%)	14.3	0.027
Miscellaneous	0 (0.0%)	4 (9.8%)	18 (5.1%)	0 (0.0%)	0 (0.0%)	1 (9.1%)	1 (6.7%)	8.13	0.229
Nerve/neurologic complication	0 (0.0%)	0 (0.0%)	26 (7.4%)	1 (4.2%)	1 (1.6%)	3 (27.3%)	0 (0.0%)	16.8	0.01
Pain or discomfort	2 (15.4%)	4 (9.8%)	70 (19.8%)	2 (8.3%)	8 (12.7%)	7 (63.6%)	5 (33.3%)	22.3	0.001
Patient reaction	0 (0.0%)	9 (22.0%)	19 (5.4%)	1 (4.2%)	3 (4.8%)	0 (0.0%)	0 (0.0%)	21.0	0.002
Peri-implant fracture	5 (38.5%)	2 (4.9%)	58 (16.4%)	1 (4.2%)	6 (9.5%)	0 (0.0%)	3 (20.0%)	15.8	0.015
Soft-tissue injury	3 (23.1%)	0 (0.0%)	32 (9.1%)	3 (12.5%)	9 (14.3%)	0 (0.0%)	0 (0.0%)	12.2	0.057
Vascular complication	0 (0.0%)	0 (0.0%)	3 (0.8%)	1 (4.2%)	0 (0.0%)	0 (0.0%)	0 (0.0%)	4.77	0.574
Total	13 (100.0%)	41 (100.0%)	353 (100.0%)	24 (100.0%)	63 (100.0%)	11 (100.0%)	15 (100.0%)	-	-

Across manufacturers, the distribution of reported device problem categories differed for hardware breakage (p=0.004), incompatibility/mismatches (p<0.001), installation/positioning (p<0.001), and stability issues (p<0.001) (Table 3). These differences reflect heterogeneity in the categories reported within MAUDE and should not be interpreted as comparative device performance or manufacturer-related causality.

Device events by locking mechanism

Among reports with device problem codes and a specified locking mechanism (Table 4), fixed-angle (n=30) most frequently reported hardware breakage (13; 43.3%), incompatibility (5; 16.7%), and material problems (5; 16.7%). Variable-angle (n=228) reported hardware breakage (78; 34.2%), incompatibility (52; 22.8%), and stability problems (29; 12.7%). Less commonly reported complications are depicted in Table 4.

**Table 4:** Locking mechanism distribution of device and patient problem categories Values are presented as N (%). For device category rows, percentages are calculated out of all device-related problems for each locking mechanism (variable-angle n=228, fixed-angle n=30). For patient category rows, percentages are calculated out of all patient-related problems for each locking mechanism (variable-angle n=193, fixed-angle n=81). χ² (df=1) and p-values are derived via Pearson chi-square tests comparing variable-angle with fixed-angle for each row.

	Variable-angle	Fixed-angle	χ²	p
Device category, N (%)				
Hardware breakage	78 (34.2%)	13 (43.3%)	0.970	0.326
Incompatibility mismatch	52 (22.8%)	5 (16.7%)	0.580	0.446
Stability issue	29 (12.7%)	2 (6.7)	0.920	0.338
Use of device problem	22 (9.6%)	3 (10.0%)	0.004	0.951
Material problem	20 (8.8%)	5 (16.7%)	1.89	0.169
Installation/positioning	13 (5.7%)	0 (0%)	1.80	0.18
Defective device	8 (3.5%)	1 (3.3%)	0.002	0.961
Difficulty advancing	5 (2.2%)	1 (3.3%)	0.150	0.697
Miscellaneous	1 (0.4%)	0 (0%)	0.130	0.716
Total	228 (100%)	30 (100%)	-	-
Patient category, N (%)				
Device issue with no consequence to patient	37 (19.2%)	4 (4.9%)	9.08	0.003
Pain or discomfort	31 (16.1%)	18 (22.2%)	1.47	0.225
Loss of range of motion or implant failure	29 (15.0%)	7 (8.6%)	2.04	0.153
Peri-implant fracture	26 (13.5%)	13 (16.0%)	0.310	0.577
Soft-tissue injury	22 (11.4%)	8 (9.9%)	0.140	0.713
Healing/recovery issue	18 (9.3%)	3 (3.7%)	2.55	0.11
Patient reaction	15 (7.8%)	4 (4.9%)	0.710	0.399
Infection	5 (2.6%)	6 (7.4%)	3.44	0.064
Miscellaneous	4 (2.1%)	8 (9.9%)	8.30	0.004
Nerve/neurologic complication	3 (1.6%)	9 (11.1%)	12.4	<0.001
Device embedment/calcification	2 (1.0%)	1 (1.2%)	0.020	0.886
Vascular complication	1 (0.5%)	0 (0%)	0.420	0.516
Total	193 (100%)	81 (100%)	-	-

There were no significant differences in the distribution of reported device events between fixed-angle and variable-angle locking plates (Table 4).

Device events by plate subtype

Column-based plates (n=94) most frequently reported incompatibility (31; 33.0%), hardware breakage (26; 27.7%), and use of device problems (13; 13.8%). Narrow plates (n=52) reported incompatibility (17; 32.7%), followed by hardware breakage (10; 19.2%) and stability issues (7; 13.5%). Short plates (n=17) were led by material problems (4; 23.5%) and stability issues (4; 23.5%), followed by hardware breakage (3; 17.7%). Extended/long plates (n=13) most often involved material problems (5; 38.5%), hardware breakage (4; 31.1%), and use of device issues (3; 23.1%). Further subtype details are provided in Table 5.

**Table 5:** Plate design distribution of device and patient problem categories Values are presented as N (%). For device category rows, percentages are calculated out of all device-related problems for each plate design (column-based n=94; extended/long n=13; extraarticular n=4; fragment-specific n=0; narrow n=52; shape-specific n=4; short n=17; spanning plates n=0; T-/L-shape n=10; wide n=5). For patient category rows, percentages are calculated out of all patient-related problems for each plate design (column-based n=64; extended/long n=13; extraarticular n=6; fragment-specific n=1; narrow n=52; shape-specific n=9; short n=18; spanning plates n=2; T-/L-shape n=11; wide n=8). χ² (df=7 for device; df=9 for patient) and p-values are derived via Pearson chi-square tests comparing distributions across plate design reports.

	Column-based	Extended/long	Extraarticular	Fragment-specific	Narrow	Shape-specific	Short	Spanning plates	T-/L-shape	Wide	χ²	p
Device category, N (%)												
Defective device	2 (2.1%)	0 (0.0%)	0 (0.0%)	0 (0.0%)	3 (5.8%)	0 (0.0%)	1 (5.9%)	0 (0.0%)	0 (0.0%)	1 (20.0%)	6.72	0.458
Difficulty advancing	1 (1.1%)	0 (0.0%)	0 (0.0%)	0 (0.0%)	0 (0.0%)	0 (0.0%)	1 (5.9%)	0 (0.0%)	1 (10.0%)	0 (0.0%)	8.37	0.301
Hardware breakage	26 (27.7%)	4 (30.8%)	3 (75.0%)	0 (0.0%)	10 (19.2%)	0 (0.0%)	3 (17.6%)	0 (0.0%)	7 (70.0%)	0 (0.0%)	20.0	0.006
Incompatibility mismatch	31 (33.0%)	1 (7.7%)	0 (0.0%)	0 (0.0%)	17 (32.7%)	1 (25.0%)	0 (0.0%)	0 (0.0%)	0 (0.0%)	0 (0.0%)	18.8	0.009
Installation/positioning	4 (4.3%)	0 (0.0%)	0 (0.0%)	0 (0.0%)	2 (3.8%)	1 (25.0%)	2 (11.8%)	0 (0.0%)	1 (10.0%)	3 (60.0%)	29.2	<0.001
Material problem	9 (9.6%)	5 (38.5%)	1 (25.0%)	0 (0.0%)	6 (11.5%)	0 (0.0%)	4 (23.5%)	0 (0.0%)	0 (0.0%)	0 (0.0%)	13.9	0.053
Miscellaneous	0 (0.0%)	0 (0.0%)	0 (0.0%)	0 (0.0%)	1 (1.9%)	0 (0.0%)	0 (0.0%)	0 (0.0%)	0 (0.0%)	1 (20.0%)	20.0	0.005
Stability issue	8 (8.5%)	0 (0.0%)	0 (0.0%)	0 (0.0%)	7 (13.5%)	1 (25.0%)	4 (23.5%)	0 (0.0%)	0 (0.0%)	0 (0.0%)	8.90	0.26
Use of device problem	13 (13.8%)	3 (23.1%)	0 (0.0%)	0 (0.0%)	6 (11.5%)	1 (25.0%)	2 (11.8%)	0 (0.0%)	1 (10.0%)	0 (0.0%)	3.26	0.859
Total	94 (100.0%)	13 (100.0%)	4 (100.0%)	0 (0.0%)	52 (100.0%)	4 (100.0%)	17 (100.0%)	0 (0.0%)	10 (100.0%)	5 (100.0%)	-	-
Patient category, N (%)												
Device embedment/calcification	0 (0.0%)	0 (0.0%)	0 (0.0%)	0 (0.0%)	1 (1.9%)	0 (0.0%)	0 (0.0%)	0 (0.0%)	0 (0.0%)	0 (0.0%)	2.55	0.979
Device issue with no consequence to patient	17 (26.6%)	2 (15.4%)	1 (16.7%)	0 (0.0%)	11 (21.2%)	2 (22.2%)	4 (22.2%)	0 (0.0%)	3 (27.3%)	2 (25.0%)	2.17	0.989
Healing/recovery issue	4 (6.2%)	2 (15.4%)	0 (0.0%)	0 (0.0%)	3 (5.8%)	0 (0.0%)	2 (11.1%)	1 (50.0%)	0 (0.0%)	0 (0.0%)	11.0	0.276
Infection	2 (3.1%)	0 (0.0%)	0 (0.0%)	1 (100.0%)	2 (3.8%)	1 (11.1%)	0 (0.0%)	0 (0.0%)	0 (0.0%)	0 (0.0%)	33.4	p<0.001
Loss of range of motion or implant failure	8 (12.5%)	5 (38.5%)	1 (16.7%)	0 (0.0%)	7 (13.5%)	0 (0.0%)	5 (27.8%)	0 (0.0%)	0 (0.0%)	2 (25.0%)	12.9	0.169
Miscellaneous	2 (3.1%)	0 (0.0%)	0 (0.0%)	0 (0.0%)	3 (5.8%)	1 (11.1%)	0 (0.0%)	0 (0.0%)	0 (0.0%)	0 (0.0%)	4.79	0.852
Nerve/neurologic complication	1 (1.6%)	0 (0.0%)	1 (16.7%)	0 (0.0%)	0 (0.0%)	1 (11.1%)	0 (0.0%)	0 (0.0%)	1 (9.1%)	1 (12.5%)	14.1	0.118
Pain or discomfort	8 (12.5%)	1 (7.7%)	2 (33.3%)	0 (0.0%)	6 (11.5%)	2 (22.2%)	3 (16.7%)	0 (0.0%)	4 (36.4%)	1 (12.5%)	7.97	0.537
Patient reaction	2 (3.1%)	0 (0.0%)	0 (0.0%)	0 (0.0%)	6 (11.5%)	0 (0.0%)	1 (5.6%)	0 (0.0%)	0 (0.0%)	0 (0.0%)	7.96	0.539
Peri-implant fracture	14 (21.9%)	1 (7.7%)	1 (16.7%)	0 (0.0%)	10 (19.2%)	2 (22.2%)	0 (0.0%)	0 (0.0%)	2 (18.2%)	1 (12.5%)	6.71	0.668
Soft-tissue injury	6 (9.4%)	2 (15.4%)	0 (0.0%)	0 (0.0%)	3 (5.8%)	0 (0.0%)	3 (16.7%)	1 (50.0%)	1 (9.1%)	1 (12.5%)	8.21	0.513
Total	64 (100.0%)	13 (100.0%)	6 (100.0%)	1 (100.0%)	52 (100.0%)	9 (100.0%)	18 (100.0%)	2 (100.0%)	11 (100.0%)	8 (100.0%)	-	-

Across plate subtypes, the distribution of reported device problem categories differed for hardware breakage (p=0.006), incompatibility/mismatch (p=0.009), and installation/positioning (p<0.001) (Table 5).

Patient adverse events

Among the total reported patient-related complications (n=520), pain or discomfort was most frequently reported (98; 18.8%), followed by peri-implant fracture (75; 14.4%), loss of ROM or implant failure (64; 12.3%), soft-tissue injury (47; 9.0%), healing/recovery issue (40; 7.7%), patient reaction (32; 6.2%), nerve/neurologic complication (31; 6.0%), and infection (28; 5.4%). Less commonly reported events such as calcification, device embedment in tissue, and vascular complications are detailed in Table 2.

Patient events by manufacturer

DePuy Synthes (n=353) most frequently cited patient pain or discomfort (70; 19.8%), peri-implant fracture (58; 16.4%), soft-tissue injury (32; 9.1%), infection (27; 7.6%), and nerve/neurologic complication (26; 7.4%). Among Stryker cases (n=63), loss of ROM or implant failure was the most frequently reported patient complications (15; 23.8%), followed by soft-tissue injury (9; 14.3%), pain or discomfort (8; 12.7%), healing and recovery issue (7; 11.1%), and peri-implant fracture (6; 9.5%). Arthrex cases (n=41) most frequently reported device embedment/calcification (9; 22.0%), patient reaction (9; 22.0%), loss of ROM or implant failure (8; 19.5%), and healing/recovery issue (5; 12.2%). Smith+Nephew (n=24) was led by loss of ROM or implant failure (4; 16.7%), healing/recovery issue (3; 12.5%), and soft-tissue injury (3; 12.5%). Complete manufacturer reports are detailed in Table 3.

Across manufacturers, the distribution of reported patient problem categories differed for device embedment/calcification (p<0.001), loss of ROM or implant failure (p=0.027), nerve/neurologic complication (p=0.010), pain/discomfort (p=0.001), patient reaction (p=0.002), and peri-implant fracture (p=0.015) (Table 3). These differences reflect heterogeneity in the categories reported within MAUDE and should not be interpreted as comparative device performance or manufacturer-related causality.

Patient events by locking mechanism

Fixed-angle (n=81) locking mechanism most commonly involved pain or discomfort (18; 22.2%), peri-implant fracture (13; 16.0%), nerve/neurologic complication (9; 11.1%), and soft-tissue injury (8; 9.9%). Variable-angle (n=193) locking mechanism showed a similar pattern, most commonly involving pain or discomfort (31; 16.1%), followed by loss of ROM or implant failure (29; 15.0%), peri-implant fracture (26; 13.5%), and soft-tissue injury (22; 11.4%). Less commonly reported complications are depicted in Table 4.

Significant differences in patient problem categories by locking mechanism were observed for nerve/neurologic complication codes (p<0.001) (Table 4).

Patient events by plate subtype

Column-based plates (n=64) most commonly reported peri-implant fracture (14; 21.9%), loss of ROM or implant failure (8; 12.5%), and pain or discomfort (8; 12.5%). Among narrow plates (n=52), the leading complications were peri-implant fracture (10; 19.2%), loss of ROM or implant failure (7; 13.5%), and patient reaction (6; 11.5%). Short plates (n=18) were led by loss of ROM or implant failure (5; 27.8%), pain or discomfort (3; 16.7%) and soft-tissue injury (3; 16.7%). Extended/long plates (n=13) most often exhibited loss of ROM or implant failure (5; 38.5%), healing/recovery issue (2; 15.4%), and soft-tissue injury (2; 15.4%). Additional subtype-specific patterns are outlined in Table 5.

Across plate subtypes, the distribution of infection differed significantly (p<0.001) (Table 5).

## Discussion

This study offers valuable insights into the most frequently reported complications and device-related failures associated with DRP systems. A total of 582 unique reports, encompassing 414 device-related and 520 patient-related codes, were analyzed. Mechanical failure of implants, most commonly breakage, fracture, or crack, emerged as the predominant device issue, while pain and discomfort were the most frequent patient-related adverse events. Given MAUDE’s passive reporting structure and limited clinical detail, the observed findings should be interpreted as a summary of commonly reported problems rather than confirmed mechanisms of failure. Yet, the findings underscore the ongoing challenge in orthopedic implant design: achieving fixation that can withstand repetitive mechanical loading while minimizing soft-tissue irritation and biologic complications [11]. The predominance of hardware breakage codes indicates that mechanical failure is a frequent theme in MAUDE reports involving DRP systems, highlighting persistent mechanical vulnerabilities in DRPs, particularly in high-stress loading and osteoporotic bone, where implant fatigue and screw-plate interface integrity remain unresolved despite the introduction of locking technology [15]. Similarly, incompatibility mismatches emphasize that implants may not always conform to variable anatomy, reinforcing the importance of intraoperative contouring, implant selection, and surgical experience. Stability problems, including migration, loosening, and detachment, along with positioning failures, highlight the interplay between surgical technique, instrumentation, and device design. Material-related issues further suggest that alloy composition, plate thickness, and geometry influence susceptibility to deformation [16]. Taken together, these reports likely reflect a combination of device factors, instrumentation, surgeon technique, and case selection.

Manufacturer stratification demonstrated distinct hypothesis-generating patterns. DePuy Synthes reports, consisting of 67.2% of all reports, most commonly consisted of device breakage and incompatibility, possibly reflecting both widespread use and potential design limitations, not to be interpreted as higher complication rates. Arthrex reports included both breakage and positioning failures, with a higher proportion of positioning issues than other manufacturers, possibly reflecting implant geometry or instrumentation complexity. Stryker and Smith+Nephew reports more frequently consisted of stability and installation problems, suggesting differences in screw-plate mechanics between manufacturers. While causality cannot be established, these findings identify possible targets for design optimization and clinical refinement to potentially reduce future complications.

Patient-related codes demonstrated patterns that highlight important clinical considerations in DRP fixation. Pain and discomfort were the most common concerns in the observed findings and are often explained by hardware prominence, tendon irritation, or altered wrist biomechanics in prior studies [17]. The occurrence of peri-implant fractures illustrates the paradox of rigid fixation, since restoring alignment may simultaneously create stress concentrations at the bone-implant interface that predispose to secondary injury [10]. Functional loss is particularly significant because it reflects both mechanical failure and biologic sequelae such as stiffness, scar tissue formation, or malunion, each of which can have long-term consequences for patient quality of life [18]. Less frequent but clinically important complications included soft-tissue injury, neuropathy, and hypersensitivity reactions [10]. While uncommon overall, the reported hypersensitivity reactions may raise awareness that biologic intolerance to implant materials may be relevant in certain patient populations. In total, these complications highlight the vulnerability of tendons and nerves in the confined anatomy of the distal forearm. Variation across manufacturers is unlikely to represent intrinsic device inferiority but instead reflects the combined influence of design features, surgical technique, and patient-specific factors [19]. Taken together, these findings reinforce that patient complications emerge at the intersection of mechanical design and biologic response and should be anticipated through careful implant selection, attention to soft-tissue corridors and screw placement, and structured postoperative rehabilitation aimed at preserving function [20].

Locking mechanism types appeared to influence patterns of complications in ways that reflect the mechanical principles of each construct. Fixed-angle plates provided rigid stability but seemed more prone to abrupt failure, which may be explained by concentrated stress at the plate-bone interface when subjected to repetitive loading [6]. In contrast, variable-angle plates allowed greater intraoperative flexibility, yet this adaptability may have introduced complexity that manifested as positioning errors, reduced stability, or difficulties in achieving precise alignment. Subtype analysis further emphasized that design features shape outcomes [21]. Column-based plates were more often limited by difficulties conforming to variable anatomy, narrow plates appeared vulnerable to loss of mechanical strength, extended plates were more likely to deform under bending stresses, and short plates often carried compromises in both stability and material strength. These patterns suggest that each design not only offers advantages but also introduces inherent trade-offs between rigidity, anatomic conformity, and soft-tissue preservation [22]. For the surgeon, these observations reinforce the importance of matching implant type to fracture morphology, patient anatomy, and functional demands, while recognizing that no plate design eliminates risk entirely.

Significant heterogeneity in adverse event profiles by manufacturer, locking mechanism, and plate subtype was identified, mainly surrounding hardware breakage, installation/positioning, pain, neurologic/nerve, and infection. The observed patterns may reflect specific design features, manufacturing, instrumentation, or clinical indications for which specific constructs are chosen. The heterogeneity between these domains may help prioritize topics for postmarket surveillance, surgeon awareness, and future denominator-based comparative studies; though, they should not be used to infer comparative safety or to make manufacturer-specific clinical recommendations.

Taken together, these findings suggest important implications for both surgeons and manufacturers. Implant design should continue to evolve toward balancing mechanical robustness with anatomic conformity, through improved contouring options, advanced alloys, and intuitive locking mechanisms. Surgeons must weigh implant selection not only by fracture morphology but also by patient-specific factors such as bone quality, soft-tissue envelope, and activity level [23]. Careful preoperative planning, precise intraoperative technique, and attention to implant positioning remain critical to mitigate complications such as peri-implant fractures, nerve injury, and soft-tissue irritation. Postoperative surveillance and rehabilitation should anticipate common complications, including pain, functional loss, and fracture recurrence, in order to optimize long-term outcomes [24].

As with all MAUDE-based studies, several limitations should be acknowledged [25]. Reports are subject to underreporting, reporting bias, variable quality of detail, and true incidence rates or comparative risk cannot be determined, given the absence of denominator and contextual data. Important clinical information, such as fracture type, patient comorbidities, or surgical technique, was often missing, limiting interpretation. Because implant choice is influenced by individual fracture complexity, surgeon preference/experience, and intraoperative decision-making, confounding by indication and technique is possible and limits any manufacturer- or design-level interpretations. Device characteristics were not consistently documented or lacked clear definitions, introducing a reliance on subjective interpretation of a reported device’s characteristics. Although we attempted to mitigate this by only strictly including devices with clear descriptors, the possibility of any subjective influence cannot be ruled out. Furthermore, the predominance of any specific manufacturer reports may reflect market share rather than higher complication rates. These results, therefore, represent observational patterns of association and cannot be used to infer comparative safety, incidence, or causality, and should be approached cautiously and only as observational data. Prospective, multicenter studies are warranted to validate these findings and refine strategies to improve implant design and optimize patient care. Lastly, because problem-code categories were defined a priori by collapsing semantically similar MAUDE terms, some misclassification is possible, and category definitions may differ across studies. Despite these limitations, this analysis presents valuable information on the potential complications and adverse events associated with DRP systems and extends the literature on these devices.

## Conclusions

In this national analysis of MAUDE reports for DRP systems (2018-2024), hardware breakage emerged as the leading device-related event, and pain/discomfort as the most common patient-related event. Adverse-event profiles differed by manufacturer and plate subtype, notable for hardware breakage, incompatibility/mismatch, neurologic complications, and infection; however, these patterns should be interpreted as observational patterns and associations, rather than comparative risk. Locking mechanisms (fixed vs. variable-angle) have broadly similar complication distributions, aside from neurologic complications. In total, the observed findings highlight enduring trade-offs among devices that may help contextualize commonly reported problem categories and inform priorities for surveillance and future denominator-based studies. Given the inherent limitations of MAUDE, true incidence and comparative risk cannot be inferred, and these findings should not be interpreted as recommendations for or against any specific manufacturer. Prospective, multicenter studies and registry-based analyses with exposure denominators are warranted to validate these findings.
